# Editorial: Innovations in supportive care in global pediatric oncology

**DOI:** 10.3389/fonc.2026.1793967

**Published:** 2026-02-06

**Authors:** C. Nathan Nessle, Ana Patricia Alcasabas, Miguela A. Caniza, Festus Njuguna, Nmazuo W. Ozuah, Terry A. Vik

**Affiliations:** 1 Department of Pediatrics, Division of Pediatric Hematology-Oncology, University of Michigan, Ann Arbor, MI, United States; 2 Cancer Institute and Pediatric Hematology-Oncology, University of the Philippines-Philippines General Hospital, Manila, Philippines; 3 Global Pediatric Medicine, Infectious Disease, St. Jude Children’s Research Hospital, Memphis, TN, United States; 4 Department of Child Health and Pediatrics, Moi University, Eldoret, Kenya; 5 Department of Pediatrics, Baylor College of Medicine, Houston, TX, United States; 6 Academic Model for Providing Access to Healthcare, Eldoret, Kenya; 7 Department of Pediatrics, Division of Hematology-Oncology, Riley Hospital for Children, Indiana University School of Medicine, Indianapolis, IN, United States

**Keywords:** childhood cancer, communication, fever, implementation science, infection, family, team science

Over 90% of children diagnosed with cancer live in low- and middle-income countries (LMICs) where the cure rate is under 30% compared to over 85% in high-income countries (1, 2). The health disparities contributing to the poor outcomes in LMICs are multifactorial and closely linked to supportive care resources. Since these resources are often limited in LMICs, oncologists must use adapted, reduced-intensity regimens to mitigate the effect of suboptimal supportive care and the risk of treatment-related mortality. Supportive care encompasses broad, multidisciplinary services that focus on preventing and managing treatment side effects, promoting adherence to therapy, and optimizing health outcomes at all stages of the cancer journey (3, 4). Effective measures to enhance the delivery of supportive care improve survival and decrease the disease burden in children with cancer (2, 5).

However, developing and implementing supportive care measures can be time-consuming, involve a deep understanding of local healthcare systems, and require access to appropriate resources, which often pose challenges for oncologists in LMICs. This Research Topic highlights supportive care strategies that emphasize contextual factors, innovative approaches, and adaptive interventions designed to address significant gaps in cancer care delivery. The studies cover a range of topics, from communication practices to infection prevention and control programs. This Research Topic emphasizes the increased use of implementation science techniques to advance care for children with cancer in these settings. The series includes 6 articles from 53 authors (25 LMIC authors), and an LMIC contributor served as first or senior author for every publication.

Baker et al. focus their message on improving outcomes for children with brain tumors in LMICs. They recognize that effective management of this population requires a multidisciplinary team of highly skilled subspecialists (e.g., neuroradiology, neurosurgery, palliative care, radiation oncology) and robust supportive resources. Optimal performance of this team can be achieved through a centralized, tertiary referral center coordinated by a formalized pediatric neuro-oncology program. Effectively implemented pediatric neuro-oncology programs should be continuously monitored to ensure the balance of care improvement with new challenges that may arise, requiring tailored readjustments to such programs.

Kisembe et al. identify factors within the hospital system that act as barriers to effective fever management for hospitalized children with cancer in Kenya. In their qualitative study, they highlight the perspectives of healthcare providers, finding that empowering healthcare workers and caregivers, timely fever management, and the shortage of human resources were major themes. While most barriers were within the hospital system, healthcare provider characteristics are key facilitators for management. Importantly, their analysis identifies strategies to minimize hospital system barriers to improve acute fever management and multidisciplinary team function.

Kipchumba et al. evaluate patient outcomes in a study of febrile neutropenic (FN) and non-neutropenic febrile (NNF) episodes. Their team describes significant delays in fever management milestones, identifies risk factors associated with poor outcomes, and notes more deaths in NNF episodes. However, they recognize that risk factors may shift as efforts to improve care delivery and fever management in an LMIC setting progress, and do not recommend using risk stratification to guide fever management. This study also highlights the difficulties in adapting the interpretation and application of research studies conducted in a high-income setting to an LMIC setting; the FN guidelines recommend a risk-stratified approach (6), and recent NNF evidence suggests a majority of episodes may not need empiric antibiotic administration (7).

Acebo et al. provide a pragmatic sustainability model in their description of establishing infection control and prevention programs at two centers in Latin America. This study provides a detailed account of a large-scale initiative, systematically guiding the readers through every stage, from the initial formation of the collaborative teams and strategies for local expansion to the consideration of performance metrics. The authors describe their approach to training and equipping local teams, implementing ongoing surveillance, and using key performance indicators to track outcomes, including specific infection rates. Importantly, their model provides a clear path to long-term sustainability, leading to a decrease in healthcare-associated infection rates and improved patient outcomes.

Facilitating communication is emphasized in Graetz et al.’s publication amongst caregivers of children with cancer in Pakistan. Their validated, mixed-methods survey reveals that over 90% of caregivers recognize the importance of effective communication from the healthcare team across topics such as shared decision-making, relationship building, coping, and informational exchange. They emphasize that caregivers of children with cancer have high information needs. Many caregivers identified communication gaps, highlighting opportunities to improve patient-centered care models through better communication.

Zeng et al. report on factors that affect family functioning among children with retinoblastoma. They note that families of children with retinoblastoma express increased social needs. The participants with the highest skills in family management had high social support systems and less extensive disease, while those in the lowest category were more likely to have stress, depression, or a lower level of education. Effective communication strategies and supporting the comprehensive needs of families with low literacy levels or a lack of social resources are strategies to improve care delivery.

Collectively, these articles underscore the gaps in supportive care in LMICs. Oncologists in these regions frequently face a multifactorial combination of health system challenges (2), social determinants (8), and treatment-related toxicities (9). Despite these challenges, the articles present efforts to improve care delivery by identifying and implementing various strategies tailored to overcome barriers. Implementation science has gained traction as a useful skill set to increase clinical uptake of evidence-based practices (10), and there is an urgent need for expanding implementation science efforts in pediatric oncology (11), particularly in an LMIC setting (12). While these studies were conducted in diverse settings across the globe, the implementation science principles and recommended strategies to improve cancer care delivery from each article are likely applicable in many other LMIC centers: 1) identify gaps in care delivery and their contributing barriers, 2) use local evidence to tailor a strategy or intervention to the barriers, then 3) implement programs with sustainability in mind to improve cancer care delivery.
